# Antiangiogenic Herbal Composition Ob-X Reduces Abdominal Visceral Fat in Humans: A Randomized, Double-Blind, Placebo-Controlled Study

**DOI:** 10.1155/2018/4381205

**Published:** 2018-05-28

**Authors:** Jae-Heon Kang, In Sun Jeong, Min-Young Kim

**Affiliations:** ^1^Inje University Seoul Paik hospital, Seoul 04551, Republic of Korea; ^2^AngioLab, Inc., Daejeon 34016, Republic of Korea

## Abstract

Adipose tissue growth is angiogenesis-dependent, and angiogenesis inhibitors can regulate adipose tissue mass by cutting off the blood supply. We examined whether antiangiogenic herbal composition Ob-X can reduce fast-growing abdominal fat, especially visceral fat in humans by inhibiting angiogenesis. Eighty abdominally obese subjects (body mass index: 25-29.9 kg/m^2^, waist circumference: exceeding 90 cm for males and 85 cm for females) participated in a 12-week randomized, double-blind, placebo-controlled human study to evaluate the efficacy and safety of Ob-X. 690 mg of Ob-X was administered orally twice a day. The Ob-X group showed a noticeable reduction in visceral fat of 20.5% after the 12-week treatment as compared to baseline measured by computed tomography. The change in visceral fat in the Ob-X group was statistically significant as compared to the placebo group (p = 0.0495) and 1.9 times higher than in the placebo group. Therefore, angiogenesis inhibitor Ob-X has the potential to improve obesity-related metabolic syndrome by reducing dangerous visceral fat.

## 1. Introduction

Obesity, particularly abdominal obesity, plays an important role in the pathogenesis of metabolic disorders and cardiovascular diseases, including type 2 diabetes [1, 2], hypertension [3, 4], dyslipidemia [5], and coronary artery disease [6]. Accumulated fat in the intra-abdominal or visceral depots is closely associated with all obesity-related complications, and it has become quite clear that visceral adiposity is more closely associated with obesity-related complications than total adiposity [7–10]. Therefore, decreasing visceral fat is important in preventing or improving obesity-related metabolic diseases.

Angiogenesis is the process of new blood vessel formation from preexisting vasculature. Similar to tumor tissue, adipose tissue growth is angiogenesis-dependent, and adipose tissue mass can be regulated by its vasculature [11–13]. It has been shown that angiogenesis modulates adipogenesis and obesity [14]. Thus, adipose tissue angiogenesis may be a therapeutic target for obesity and metabolic disease [15].

We screened antiangiogenic and MMP-inhibitory activities from herbal extract libraries and found that three herbal extracts of* Morus alba *L. (Moraceae; white mulberry),* Melissa officinalis *L. (Lamiaceae; lemon balm), and* Artemisia capillaris *Thunb. (Compositae; Injin or Yin Chen Hao) showed antiangiogenic and MMP-inhibitory activities [16]. Moreover, the combination of three herbal extracts showed synergistic effect in inhibiting angiogenesis, compared with each extract alone.

Melissa has been used as a medicinal plant to treat nervousness, insomnia, gastrointestinal disorders, herpes virus infection, and Alzheimer's disease [17, 18]. It is well known that aqueous or aqueous ethanolic extract of Melissa contains phenolic compounds. The prominent phenolic compound in Melissa extract is rosmarinic acid, which has a number of interesting biological activities, such as antiviral, antibacterial, antiinflammatory, antioxidant [19], and antiangiogenic [20]. Aqueous white mulberry extract contains 1-deoxynojirimycin, which is known to be one of the most potent *α*-glycosidase inhibitors [21]. A water extract of* Artemisia capillaris *had protective effects against oxidative stress induced liver damage in rats [22]. Each herbal extract was standardized with a reference compound to maintain the quality.

In animal studies, a mixture of three herbal extracts, called Ob-X, reduced adipose tissue mass in high fat diet-induced obese mice, providing evidence that adipose tissue growth can be inhibited by angiogenesis inhibitor [23]. In addition, the regulation of adipose tissue growth by Ob-X altered the expression of genes involved in angiogenesis and the MMP system [23].

Ob-X supplementation in high-fat diet-induced obese mice increased the hepatic mRNA levels of the PPAR*α* target enzymes responsible for fatty acid *β*-oxidation [24]. The study demonstrated that Ob-X regulates body weight gain, adipose tissue mass, and lipid metabolism, partly through changes in the expression of hepatic PPAR*α* target genes [24].

In genetically obese* ob/ob *mice, Ob-X also reduced body weight gain and visceral fat mass, without changes in weights of any other organs, demonstrating that Ob-X specifically targets adipose tissue [25]. Ob-X suppresses adipogenesis in 3T3-L1 adipocytes by inhibiting differentiation of preadipocytes into adipocytes [26]. These events may be mediated by changes in the expression of genes involved in lipogenesis, angiogenesis, and the MMP system [26]. Thus, antiangiogenic Ob-X may provide a possible therapeutic approach to the prevention and treatment of human obesity and related disorders, by reducing adipose tissue.

In the present study, Ob-X was evaluated in humans to determine whether it reduces fast-growing abdominal fat, especially visceral fat, by inhibiting angiogenesis.

## 2. Materials and Methods

### 2.1. Subjects

Healthy volunteers, 19-50 years of age, were recruited. Informed consent was obtained from all the subjects prior to enrolling in the study. A total of 80 volunteers, with a BMI of 25-29.9 kg/m^2^ and waist circumference exceeding 90 cm for males and 85 cm for females, met the inclusion criteria and enrolled. Exclusion criteria for the study included those on drug therapy, those on a special diet, those taking dietary substitutes for weight loss, and those involved in a diet program within 3 months prior to the start of the study. Hypertensive individuals whose SBP was higher than 160 mmHg or DBP higher than 100 mmHg, those taking diuretics, diabetics taking oral hypoglycemic agents or insulin, those with active thyroid disease or hyperlipidemia, those with a history of renal, liver, pancreatic, or chronic inflammatory/infectious diseases, allergic diseases, asthma, heart failure, or malignant tumors, pregnant or lactating women, and volunteers with suspected drug or alcohol abuse or with any clinical condition rendering them unfit to participate were also excluded from the study.

### 2.2. Preparation of Ob-X

Ob-X was prepared using food grade aqueous extracts of* Morus alba* L. (white mulberry),* Melissa officinalis* L. (lemon balm), and* Artemisia capillaris *Thunb. (Injin or Yin Chen Hao). The quality of each herbal extract contained in the Ob-X was controlled by standardization with reference compounds using high-pressure liquid chromatography (HPLC). The corresponding reference compounds are 1-deoxynojirimycin (*Morus alba *L.), rosmarinic acid (*Melissa officinalis* L.), and 6,7-dimethylesculetin (*Artemisia capillaris *Thunb.). For the human study, 230 mg of Ob-X was filled in one capsule, and 230 mg of dextrin was filled in a capsule, used as placebo, at the GMP facility. The composition of Ob-X in 230 mg is 102.1mg of white mulberry leaf extract, 102.4mg of lemon balm leaf extract, and 25.5 mg of Injin extract in powdered form.

### 2.3. Study Design

Ethical approval for this human study was obtained from the Institutional Review Board at the Inje University Seoul Paik hospital (SIT 240) in 2008 as being in compliance with the Helsinki II Declaration. It was a randomized, placebo-controlled, double-blind, 12-week study. Eighty subjects were enrolled in the study and randomized to receive placebo (1.38 g of dextrin) or 1.38 g of Ob-X per day for 12 weeks. The daily dose of Ob-X was determined by the preliminary human study (data not published). 1.38 g of Ob-X contains 0.9 g of active extracts and 0.48 g of dextrin as excipient. Subjects were instructed to take 3 capsules (230 mg/capsule) twice a day in the morning and afternoon, a total of 6 capsules per day (1.38 g). The placebo capsules and Ob-X capsules were identical in appearance.

All subjects were scheduled to visit the hospital every 4 weeks from the start of the study. During the study, all subjects were counseled on diet and exercise compliance at every visit from the nutritionist. All subjects were instructed to restrict their total energy intake to 500-kcal deficit of the recommended daily calorie intake and were advised to exercise corresponding to an energy expenditure of 250 kcal per day, three times a week. All subjects were educated to fill out a food diary, and the nutritionist analyzed the food intake of three major nutrients in terms of calorie content, using the nutrition analysis program (CAN pro) at baseline and at 12 weeks.

### 2.4. Anthropometric Measurement

Baseline characteristics and demographic data were recorded by the medical staff as the subjects entered the study, to evaluate their eligibility. Body weight, height, waist and hip circumferences, BMI, and vital signs, such as systolic and diastolic blood pressure and heart rate measurements, were recorded.

### 2.5. Laboratory Measurement

White blood cell (WBC), red blood cell (RBC), hematocrit, hemoglobin, and platelet were determined as hematological studies. Creatinine, blood urea nitrogen (BUN), uric acid, calcium (Ca), potassium (P), sodium (Na), potassium (K), chloride (Cl), insulin, aspartate aminotransferase (AST), alanine aminotransferase (ALT), albumin, protein, alkaline phosphatase(ALP), total cholesterol, LDL-cholesterol, HDL-cholesterol, triglyceride, glucose, and C-reactive protein (CRP) were determined as laboratory test items for safety analysis.

### 2.6. Clinical Assessment

The primary outcome measure of this study was the reduction of abdominal fat at week 12 as compared to baseline. Computed tomography was used, on a Siemens CT scanner (Erlangen, Germany), to determine total abdominal fat, visceral fat, and subcutaneous fat areas at baseline and at week 12. An image of the cross section at the L4/L5 intervertebral disc level was obtained and was analyzed using Siemens' software.

Body composition was measured by bioelectric impedance analysis (BIA, BC-418, Tanita, Japan) at baseline and at week 12.

### 2.7. Compliance Assessment

Subjects were asked to return all unused capsules in the original bottles every 4 weeks. The returned capsules were counted and the compliance was calculated as the number of capsules actually consumed divided by the number of capsules that should have been used by the end of 12 weeks of treatment. Each subject was considered compliant when taking at least 70% of Ob-X or placebo given.

### 2.8. Statistical Analysis

The effect of Ob-X was assessed by analyzing the Ob-X treated group compared with the placebo group. The results are given as mean and standard deviation. The data were processed using the SPSS statistical program, and the significance of the difference in mean value between the placebo and the Ob-X treated group was submitted to Student's t-test. ANCOVA analysis was also conducted. If the p value was smaller than 0.05, the value was regarded as statistically significant. Correlations between initial values and changes in total abdominal, visceral, and subcutaneous fat areas were assessed using Pearson's correlation coefficient.

## 3. Results

### 3.1. Baseline Status and Compliance

**Figure 1:**
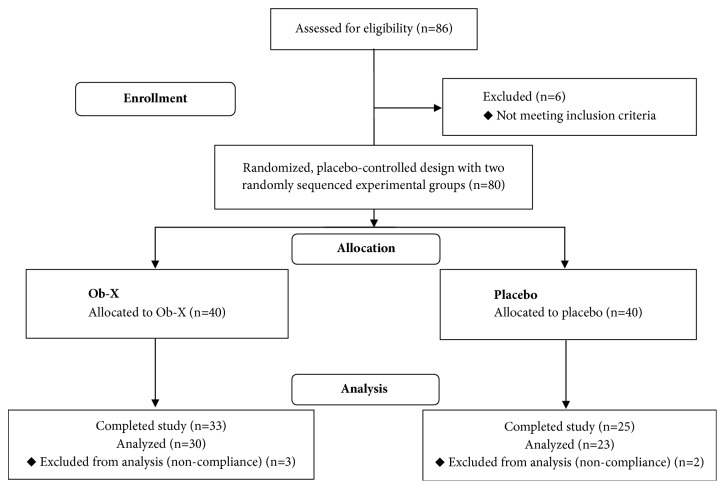
CONSORT (Consolidated Standards of Reporting Trials) flow diagram of the recruitment, enrollment, and randomization process.

As shown in Figure 1, of the total 80 subjects randomized, 58 completed the study. Of the 40 subjects in the placebo group, 25 completed the study. In the Ob-X treated group, 33 out of the 40 subjects completed the study. The noncompleters are represented by 8 subjects in the placebo group and 2 subjects in the Ob-X treated group, who discontinued the study for personal reasons; 7 subjects in the placebo group and 5 subjects in Ob-X treated group discontinued the study through loss to follow-up.

Of the 58 subjects who completed the study, 5 had compliance lower than 70% as described in the protocol. Therefore, 53 subjects completed the study according to the study protocol, and the results of these 53 subjects were analyzed, except for the safety analysis.

There were no significant differences between the characteristics of the groups at baseline, as shown in Table 1.

### 3.2. Effect of Ob-X on Abdominal Fat: Primary Outcome Measure

To determine whether Ob-X reduces fast-growing abdominal fat, visceral fat, and subcutaneous fat were assessed by computed tomography at baseline and at week 12.

**Figure 2:**
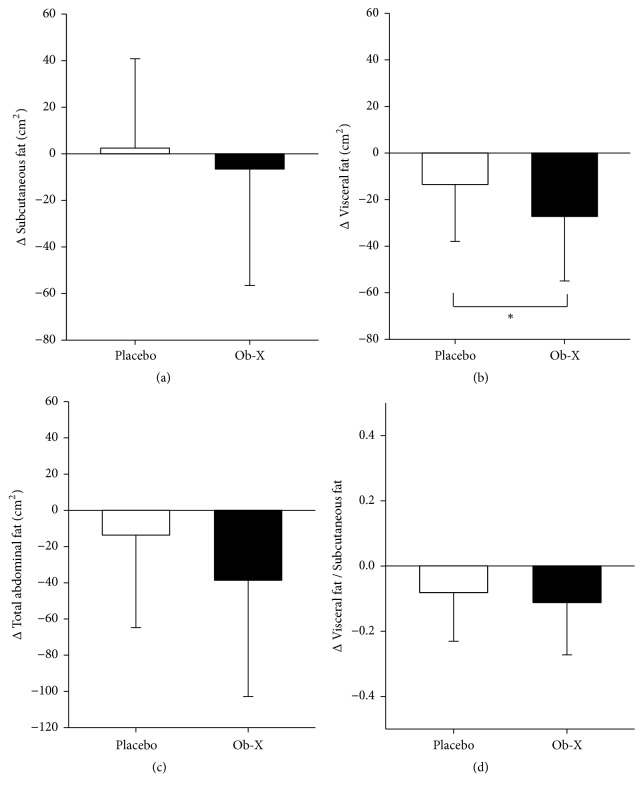
Changes in abdominal fat at week 12 of treatment with Ob-X. (a) Changes in subcutaneous fat, (b) visceral fat, (c) total abdominal fat, and (d) visceral fat/subcutaneous fat ratio (^*∗*^p < 0.05 compared with placebo).

**Figure 3:**
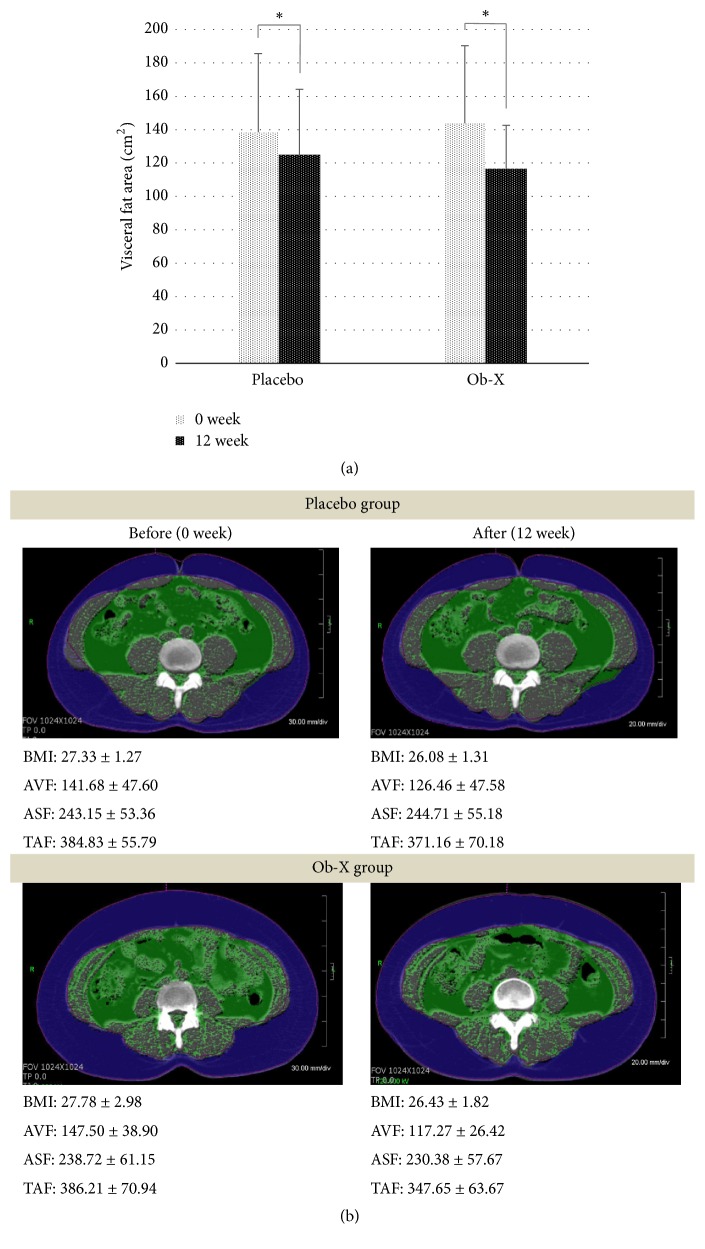
Reduction in visceral fat after 12-week treatment of Ob-X. (a) Visceral fat area was analyzed by computed tomography (^*∗*^p < 0.05 compared with placebo). (b) Representative CT-scan data at 0 and 12 weeks in a subject from Ob-X or placebo group. Differences in abdominal visceral fat (AVF), abdominal subcutaneous fat (ASF), and total abdominal fat (ATF) areas at 0 and 12 weeks were measured. Computed tomography scanning for measuring fat area was performed with subject in the supine position, at the lumbar vertebra 4 level. Visceral fat area of the patient before and after Ob-X treatment was represented by the green color. Values represent the mean ± SD.

When we analyzed the change in abdominal fat by Ob-X at week 12 compared to the placebo group, the Ob-X group reduced subcutaneous fat (-8.33 ± 51.63 cm^2^ versus 1.56 ± 39.88 cm^2^, respectively), visceral fat (-30.23 ± 27.28 cm^2^ versus -15.22 ± 24.61 cm^2^ respectively), total abdominal fat (-38.56 ± 64.24 cm^2^ versus -13.67 ± 51.13 cm^2^ respectively), and visceral/subcutaneous fat ratio (-0.14 ± 0.17 versus -0.09 ± 0.15) as shown in Table 2 and Figure 2. However, there was statistical significance only in the reduction of visceral fat. The Ob-X group showed a noticeable reduction in visceral fat of 20.5% after the 12-week treatment as compared to baseline. The change in visceral fat in the Ob-X group was statistically significant as compared to the placebo group (p = 0.0495) and 1.9 times higher than in the placebo group (10.7%) as shown in Figure 3. The reduction of visceral fat by Ob-X was not the result of the decrease in calorie intake by appetite suppression, since there were no significant differences in calorie intake and energy consumption between the placebo group and the Ob-X group at baseline and at week 12, as analyzed by nutrition analysis (data not shown).

This result indicates that the angiogenesis inhibitor, Ob-X, specifically reduces visceral fat, as angiogenesis occurs actively in fast-growing visceral fat.

### 3.3. The Correlation between Initial Values and Change of the Abdominal Fat Area

**Figure 4:**
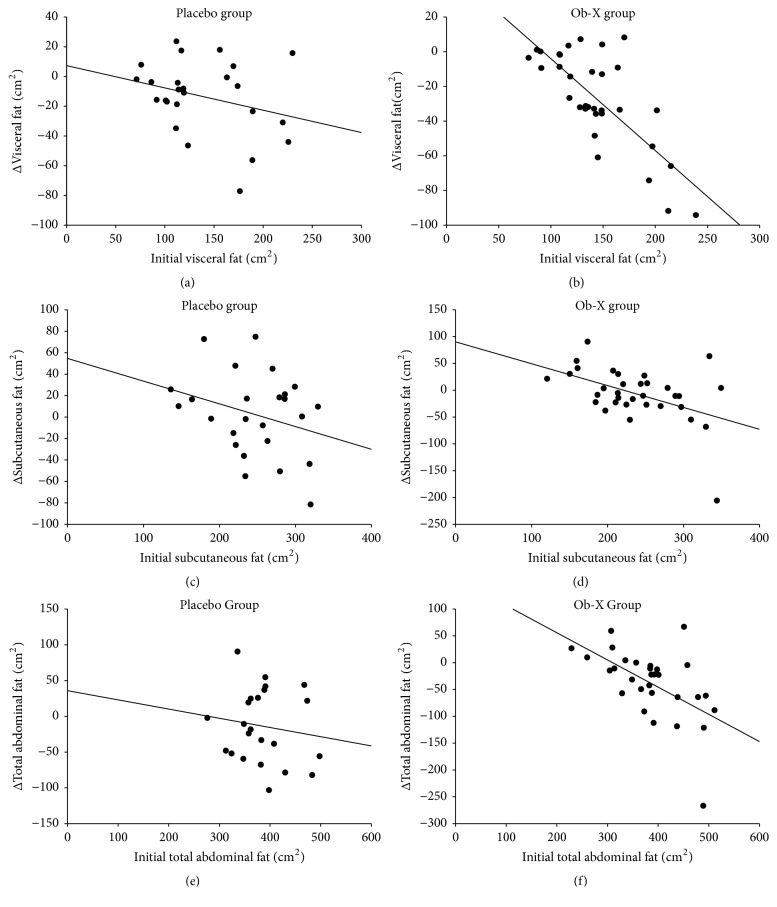
The fat reducing effect of placebo and Ob-X treatment in relation to the initial abdominal fat area. Correlation between the induced change in visceral fat and initial visceral fat before intervention of placebo (a) or Ob-X (b). Correlation between the induced change in subcutaneous fat and initial subcutaneous fat before intervention of placebo (c) or Ob-X (c). Correlation between the induced change in total abdominal fat and initial total abdominal fat before intervention of placebo (e) or Ob-X (f).

We examined the correlation between the initial visceral fat area and the change in visceral fat area induced by Ob-X treatment. As shown in Figure 4, the change in visceral fat area induced by Ob-X showed a significantly negative correlation with the initial visceral fat area (r = -0.75, p = 0.0000005). In contrast, no correlation was found between the initial visceral fat area and the placebo-induced change (r = -0.28, p = 0.16). A significantly negative correlation was also found between the initial subcutaneous fat area and the change in subcutaneous fat area induced by Ob-X (r = -0.47, p = 0.0050), but not in the placebo group (r = -0.29, p = 0.14). In addition, the change in total abdominal fat area induced by Ob-X was also negatively correlated with the initial total abdominal fat area (r = -0.56, p = 0.0012), but not in the placebo group (r = -0.14, p = 0.52).

### 3.4. Effect of Ob-X on Body Weight and Composition

Looking at the analysis data for other secondary outcome measurements, i.e., body weight, BMI, body fat composition, body fat mass, waist circumference, and WHR, there were no statistically significant differences on these variables between the placebo group and the Ob-X-treated group (Table 3). However, there was a trend towards the reduction in waist circumference and WHR in the Ob-X group. The waist circumference decreased by 4.24 ± 4.29 cm in the Ob-X treated group compared with a 3.97 ± 5.17 cm decrease in the placebo group after 12 weeks of treatment. The change in waist-to-hip ratio was -0.02 ± 0.04 in the Ob-X treated group compared with -0.01 ± 0.04 in the placebo group.

### 3.5. Serum Lipids and Others

In the serum analysis, there were no statistically significant changes between the placebo group and the Ob-X-treated group at baseline and at week 12, in terms of total cholesterol, LDL-cholesterol, HDL-cholesterol, triglyceride, glucose, and CRP, which were all in the normal ranges in both groups (data not shown).

### 3.6. Safety Analysis

Adverse events were observed during the study period in the Ob-X group and placebo group, as shown in Table 4. All these events were transient, and there was no observed aggravation due to the continuous intake of Ob-X capsules and the placebo capsules in each group. There was no difference between the groups in the incidence of self-reported adverse events.

The safety analysis for clinical laboratory test items (WBC, RBC, hematocrit, hemoglobin, platelet, creatinine, insulin, BUN, uric acid, Ca, P, Na, K, Cl, AST, ALT, albumin, protein, and ALP) and vital signs was conducted for 58 subjects (placebo: 25, Ob-X: 33), who were randomized and completed this study. There were no problematic abnormalities or findings. The mean values for all the clinical laboratory test items and vital signs from baseline to week 12 were in the normal range, and the changes were similar between the groups (data not shown). There was no statistical difference between the placebo and Ob-X groups, from baseline to week 12. Based on these results, the safety of Ob-X is similar to placebo, and it is considered that Ob-X has a good safety profile.

## 4. Discussion

Angiogenesis has been shown to play a crucial role in the modulation of adipogenesis and obesity [14, 27]. Modulators of angiogenesis affect the expansion and metabolism of fat mass by regulating the growth and remodeling of the adipose tissue vasculature [15]. Therefore, pharmacological control of adipose tissue neovascularization by angiogenesis inhibitors might offer a novel therapeutic option for the treatment of obesity and related metabolic disorders [15].

The present study demonstrated, for the first time, that the angiogenesis inhibitor Ob-X can reduce abdominal fat, especially visceral fat, in humans. Ob-X, which is composed of three herbal extracts, has synergistic effect on inhibition of angiogenesis when combined with three extracts comparing to a higher dose of a single extract [data not shown].

It is reported that vascular endothelial growth factor (VEGF) and its system account for most of the angiogenic activity in adipose tissue, making it an attractive target to reduce obesity [28–30]. Ob-X showed dose-dependent inhibition of VEGF-induced microvessel outgrowth from aortic tissue in the* ex vivo* rat aortic ring assay, indicating that Ob-X can inhibit VEGF-induced angiogenesis [23]. Furthermore, in high fat diet-induced obese mice, Ob-X treatment reduced mRNA expression of VEGF in visceral and subcutaneous fat [23].

MMPs play major roles in the extracellular matrix remodeling events associated with angiogenesis [31, 32] and adipogenesis [33]. It was demonstrated that MMPs have novel function in adipogenesis, modulating adipocyte differentiation. Therefore, MMP inhibitors may block the adipocyte differentiation process [34–36].* In vitro* and animal studies showed that Ob-X can regulate the growth and development of adipose tissue by inhibiting MMP activities [23]. Thus, the mechanism of Ob-X to reduce visceral fat in humans may be due to the inhibition of angiogenesis via the VEGF and MMP systems.

The Ob-X reduced adipose tissue mass in nutritionally obese mice and the size of adipocytes in Ob-X treated mice were markedly smaller than those in control mice. The size of visceral adipocytes was decreased by 63%, while the size of subcutaneous adipocytes was decreased by 32% in Ob-X-treated mice relative to control mice. In addition, in Ob-X-treated mice, the blood vessel density in visceral adipose tissue was lower than that of the controls, which shows that the decrease in visceral adipose tissue mass by Ob-X is the result of inhibition of angiogenesis [23].

Adipose tissue is one of the few adult tissues that can grow and regress throughout adulthood. This characteristic relies heavily on active angiogenesis, since the expansion of capillary beds is necessary for tissue growth. Accordingly, adipose tissue contains extensive capillary networks surrounding the adipocytes, which are probably induced by adipocyte-secreted growth factors and hormones [37]. Adipose tissue has been shown to synthesize and release a huge number of signaling proteins, adipokines, which are involved in regulating many cellular processes, including inflammation and angiogenesis [37, 38]. Large-size adipocytes, mainly in visceral adipose tissue, are prone to rupture, evoking an inflammatory reaction [39]. In fact, we found that the size of visceral adipocytes is larger than that of subcutaneous adipocytes in high fat diet-induced obese mice [23], and angiogenesis occurs actively in visceral adipose tissue in which blood vessels are seen much more than in subcutaneous adipose tissue in animal experiments.

In a randomized placebo-controlled double-blind human study, Ob-X reduced total abdominal fat, subcutaneous fat, and visceral fat. However, a statistically significant reduction was shown only in visceral fat compared to placebo. The percentage of reduction in visceral fat by Ob-X was 20.5% as compared to baseline (10.7%), which was 1.9 times as much as placebo group. The selective effect of Ob-X in reducing visceral fat may be attributed to the different properties of visceral fat and subcutaneous fat. Specifically, visceral fat is more sensitive to angiogenesis inhibitor, which is consistent with animal studies of Ob-X [23].

The effects of Ob-X treatment in reducing visceral fat, subcutaneous fat, and total abdominal fat were negatively correlated with the initial areas of fat before intervention. The significantly negative correlation was the strongest in visceral fat. This implies that Ob-X may have a strong effect in reducing visceral fat in individuals with high visceral fat.

Although Ob-X reduced visceral fat in a statistically significant manner compared to the placebo group, we observed no effect on the other measurements related to visceral fat loss. The reduction in body fat mass and percentage body fat measured by BIA were not statistically significant compared to placebo. This may because the percentage of visceral fat in the total body fat is small, and 85% of the subjects were women, who have a larger proportion of fat in their body mass than men and are more likely to deposit fat subcutaneously [40]. In addition, when body composition was measured by BIA, which uses electric conductivity, we could not expect accuracy, given that factors that influence water content, such as water intake, perspiration, and urination and environment of measurement, are not considered.

No side effects were observed during the study period, and there were no statistically significant differences between the two groups. The adverse events reported in the Ob-X group were common cold, heartburn, constipation, dry mouth, stomachache, menstrual irregularity, and meningitis, which were considered not to be related to Ob-X. The results for the safety analysis showed no significant differences between the placebo and Ob-X-treated groups in the clinical laboratory blood test, biochemical test, blood pressure, and pulse. All the results were within normal ranges. The safety analysis suggested that Ob-X is safe. Moreover, the good compliance and low dropout rates in the Ob-X group indicate that Ob-X can be administered for long-term use.

Many studies indicate that visceral adipose tissue, independent of obesity, is a major determinant of insulin resistance and contributes to variations in insulin sensitivity, even in healthy nonobese subjects [41]. Abdominal visceral fat is particularly harmful because it impairs insulin metabolism and promotes insulin resistance [42]. The mechanism by which visceral fat causes insulin resistance is known [43]. The expression of enzymes (e.g., lipoprotein lipase, hormone sensitive lipase, and peroxisome proliferator-activated receptor *γ*) related to lipid turnover in visceral fat increases with fat feeding in visceral fat relative to subcutaneous fat. This can enhance the flow of free fatty acids (FFA) through the portal vein to the liver, as well as to other tissues [43, 44]. FFA levels are frequently high in obese individuals, and experimental evidence suggests that high FFA and Low adiponectin plasma levels play a key role in the mechanism by which excess adiposity promotes insulin resistance [45, 46]. Medina-Urrutia et al. also demonstrated the combined effect of high abdominal visceral fat and low adiponectin levels on the risk of insulin resistance [47].

Metabolic syndrome is a group of metabolic risk factors, including abdominal obesity, high blood pressure, high triglyceride levels, low levels of high density lipoprotein (HDL) cholesterol, and high levels of fasting plasma glucose [48]. Metabolic syndrome is associated with subsequent increases in the incidence of type 2 diabetes mellitus [49], cardiovascular disease morbidity [50], and even mortality [51]. It is becoming increasingly recognized that the most prevalent form of metabolic abnormalities linked to insulin resistance is found in patients with abdominal obesity, especially those with an excess of visceral adipose tissue [52]. The abdominal obesity is an essential criterion for diagnosis of metabolic syndrome defined by the International Diabetes Federation (IDF). Ethnic-specific waist circumference cut-off points have been incorporated in the definition. The cut-off value for waist circumference to define abdominal obesity in Koreans was 90 cm in men and 85 cm in women by the Korean Society of the Study of Obesity [53], which was the inclusion criteria in this study.

Hayashi et al. showed that visceral adiposity increases the odds of hypertension in Japanese Americans, independent of other adipose depots and fasting plasma insulin [54]. In addition, visceral and central abdominal fat showed a close association with type 2 diabetes in a case-control study involving 82 type 2 diabetic and 82 age- and sex-matched nondiabetic subjects [55]. It was also shown, in another human study, that visceral adiposity is a predictor of metabolic syndrome and cardiometabolic risk factor levels. Subjects whose maximum visceral adipose tissue area was higher in the abdomen had higher LDL-cholesterol concentrations, independent of age and adiposity [56]. Thus, visceral adipose tissue should be decreased to reduce the insulin resistance and the risk of metabolic syndrome. However, visceral fat is hard to lose by exercise only. It was reported that the high amount, vigorous intensity exercise equivalent to jogging 20 miles per week for about 8 months decreased the visceral fat by 6.9% [57]. In that regard, 20.5% decrease in visceral fat by Ob-X was very efficient since it is an angiogenesis inhibitor reducing fast-growing visceral fat specifically. Although Ob-X showed the decrease in blood vessels in visceral fat in animal study, further study will be necessary to analyze the angiogenic parameters in human blood.

## 5. Conclusion

The results of the present human study demonstrate that the angiogenesis inhibitor Ob-X reduces visceral fat safely in humans. Therefore, it is expected that Ob-X would reduce the risk of metabolic syndrome by reducing dangerous visceral fat.

## Figures and Tables

**Table 1:** Characteristics of the subjects at baseline.

		**Placebo group ** **(n = 23)**	**Ob-X group ** **(n = 30)**
Sex, n (%)	Male (%)	2 (8.7%)	6 (20%)
	Female (%)	21 (91.3%)	24 (80%)

Age (y)		33.61 ± 9.47	32.30 ± 7.11

Height (cm)		159.90 ± 5.62	162.70 ± 7.74

Weight (kg)		69.99 ± 6.27	72.65 ± 8.22

BMI (kg/m^2^)		27.33 ± 1.27	27.78 ± 2.98

Waist circumference (cm)		91.04 ± 3.72	90.53 ± 3.95

Hip circumference (cm)		101.58 ± 3.85	102.14 ± 4.29

Waist to hip ratio		0.897 ± 0.033	0.887 ± 0.038

Systolic BP (mmHg)		115.65 ± 10.37	119.53 ± 11.31

Diastolic BP (mmHg)		74.70 ± 10.61	75.77 ± 8.69

Pulse (beats/min)		66.52 ± 10.92	68.10 ± 10.99

Values represent the mean ± SD.

**Table 2:** Changes in abdominal fat analyzed by computed tomography.

	**Group**	**W0**	**W12**	**W12-W0**
Subcutaneous fat (cm^2^)	Placebo	243.15 ± 53.36	244.71 ± 55.18	1.56 ± 39.88
	Ob-X	238.72 ± 61.15	230.38 ± 57.67	-8.33 ± 51.63

Visceral fat (cm^2^)	Placebo	141.68 ± 47.60	126.46 ± 47.58	-15.22 ± 24.61
	Ob-X	147.50 ± 38.90	117.27 ± 26.42	-30.23 ± 27.28_ _^*∗*^

Total abdominal fat (cm^2^)	Placebo	384.83 ± 55.79	371.16 ± 70.18	-13.67 ± 51.13
	Ob-X	386.21 ± 70.94	347.65 ± 63.67	-38.56 ± 64.24

Visc.fat / Subc. fat ratio	Placebo	0.64 ± 0.36	0.55 ± 0.30	-0.09 ± 0.15
	Ob-X	0.68 ± 0.27	0.54 ± 0.18	-0.14 ± 0.17

Values represent the mean ± SD._ _^*∗*^p < 0.05 compared with placebo.

**Table 3:** Changes from week 0 to week 12 between placebo and Ob-X treated group.

	**Group**	**W0**	**W12**	**W12-W0**
Body weight (kg)	Placebo	69.99 ± 6.27	66.83 ± 6.61	-3.17 ± 2.49
	Ob-X	72.65 ± 8.22	70.07 ± 7.75	-2.58 ± 2.97

BMI (kg/m^2^)	Placebo	27.33 ± 1.27	26.08 ± 1.31	-1.25 ± 1.01
	Ob-X	27.78 ± 2.98	26.43 ± 1.82	-1.34 ± 2.35

Body fat (%) (BIA)	Placebo	39.01 ± 4.19	36.93 ± 1.15	-2.08 ± 2.24
	Ob-X	37.24 ± 5.68	35.44 ± 6.10	-1.80 ± 2.90

Body fat mass (kg) (BIA)	Placebo	27.30 ± 3.88	24.68 ± 3.75	-2.63 ± 2.39
	Ob-X	26.91 ± 4.29	24.65 ± 4.24	-2.26 ± 2.94

Waist circumference (cm)	Placebo	91.04 ± 3.72	87.07 ± 5.86	-3.97 ± 5.17
	Ob-X	90.53 ± 3.95	86.29 ± 5.84	-4.24 ± 4.29

Hip circumference (cm)	Placebo	101.58 ± 3.85	98.43 ± 4.55	-3.14 ± 2.96
	Ob-X	102.14 ± 4.29	99.56 ± 4.05	-2.58 ± 3.13

Waist / Hip ratio	Placebo	0.90 ± 0.03	0.88 ± 0.05	-0.01 ± 0.04
	Ob-X	0.89 ± 0.04	0.87 ± 0.05	-0.02 ± 0.04

Values represent the mean ± SD. BMI, body mass index.

**Table 4:** Self-reported adverse events during 12 weeks of treatment.

	**Total number of cases**	**Adverse events (no. of case)**
Placebo	15	Esophagitis(1), Headache(1), Edema(1), Nausea(1), Low back pain(1), Enteritis(2), Menstrual irregularity(1), Cold/flu(7)

Ob-X	10	Heartburn(1), Constipation(1), Dry mouth(1), Cold/flu(3) Stomachache(2), Menstrual irregularity(1), Meningitis(1),
